# Efficacy and Pharmacokinetics of Guanidine Derivative LQOF-G2 in an In Vivo *Leishmania* (*Viannia*) *braziliensis* Model

**DOI:** 10.3390/ph19101505

**Published:** 2026-09-22

**Authors:** Klinger Antonio da Franca Rodrigues, Luana Ribeiro dos Anjos, Mércya Lopes Braga, Franciregina Silva Araújo, Maria Raisse Ribeiro de Sousa, José Wheslley Rodrigues de Lucena, Patrick Vinclair, André Colom, Eric Erdociain, Aude Gendras, Anais Giry, Eduardo René Pérez González

**Affiliations:** 1 Infectious Diseases Laboratory, Campus—Ministro Reis Velloso, Federal University of Parnaíba Delta, Parnaíba 64202-020, PI, Brazil; mercyalopesbragas2@gmail.com (M.L.B.); franci123araujo@gmail.com (F.S.A.); mariaraisse@ufpi.edu.br (M.R.R.d.S.); wheslleyrodriguess@ufdpar.edu.br (J.W.R.d.L.); 2 Center for Basic and Applied Immunology, Dom Delgado University City, Federal University of Maranhão, São Luiz 65065-545, MA, Brazil; 3 Fine Organic Chemistry Lab, School of Sciences and Technology, São Paulo State University (UNESP), Presidente Prudente 19060-080, SP, Brazil; luana.anjos@unesp.br; 4 Evotec France SAS, Campus Curie, 195 Route d’Espagne, 31036 Toulouse, France; patrick.vinclair@evotec.com (P.V.); andre.colom@evotec.com (A.C.); eric.erdociain@evotec.com (E.E.); aude.gendras@evotec.com (A.G.); anais.giry@evotec.com (A.G.)

**Keywords:** cutaneous leishmaniasis, pharmacokinetics, cytokine responses

## Abstract

**Background/Objectives**: Due to their toxicity and the limited efficacy of current therapies, Leishmaniases represent a major public health challenge. The search for new treatments is urgent. LQOF-G2 is a synthetic guanidine compound whose in vitro leishmanicidal potential has already been investigated by our group. The present study evaluates the in vivo efficacy, safety-related parameters, treatment-associated cytokine responses, and pharmacokinetic (PK) properties of the novel guanidine derivative LQOF-G2. **Methods**: Golden hamsters infected with *Leishmania* (*Viannia*) *braziliensis* were treated with LQOF-G2 (5 and 10 mg/kg, i.p.). Pharmacokinetic characterization was performed in BALB/c mice following intravenous and intraperitoneal administration. **Results**: Treatment with LQOF-G2 significantly reduced cutaneous lesion size and parasite burden at both the site of infection (up to 98.6 ± 0.8%) and in the lymph nodes (up to 99.7 ± 0.3%). No marked alterations were observed in the biochemical and hematological parameters evaluated at the tested doses. Treatment was associated with increased TNF-α and IFN-γ responses and reduced IL-10 levels following ex vivo antigen stimulation, consistent with a more pro-inflammatory/type-1-associated cytokine profile. Because murine cytokine ELISA reagents were used with golden hamster samples without independent species-specific analytical validation, these findings were considered exploratory and supportive rather than quantitatively validated evidence of an immunomodulatory mechanism. Pharmacokinetic characterization in BALB/c mice revealed moderate clearance (46 ± 4 mL/min/kg), a relatively large apparent volume of distribution (Vdss = 11 ± 1 L/kg), and a long elimination half-life (6.8 ± 0.4 h) following intravenous administration. After intraperitoneal administration, rapid absorption (Tmax = 0.25 h) and an absolute bioavailability of 44% were observed. **Conclusions**: Overall, these findings support LQOF-G2 as a candidate for further preclinical investigation in cutaneous leishmaniasis.

## 1. Introduction

Due to underreporting and inadequate management, Leishmaniases (classified as a Neglected Tropical Disease (NTD) has become a serious public health challenge in many vulnerable regions. Leishmaniases are intrinsically linked to social vulnerability, poor infrastructure, and barriers to therapeutic intervention [1]. They are caused by intracellular protozoa of the genus Leishmania, comprising more than 20 pathogenic species transmitted through the bite of infected female sandflies [2,3]. Clinically, they manifest in three main forms: visceral, cutaneous, and mucocutaneous, with visceral leishmaniasis standing out due to its severity and lethality. In the absence of timely therapeutic intervention, mortality rates can exceed 95% [4]. According to the World Health Organization (WHO), global incidence ranges from 700,000 to 1 million new cases annually, distributed across more than 90 countries [5,6].

Conventional treatments include first-line drugs (such as pentavalent antimonials) and second-line drugs (such as amphotericin B, miltefosine, and pentamidine), all of which present severe toxicity profiles. The clinical use of pentavalent antimonials is often limited by cardiotoxicity, myalgia, arthralgia, and hepatic dysfunction [7]. Similarly, amphotericin B is recognized for its marked nephrotoxicity and hepatotoxicity, in addition to inducing significant metabolic imbalances [8]. Miltefosine can cause gastrointestinal disturbances and has proven teratogenic potential, contraindicating its use during pregnancy. Additionally, the utility of paromomycin is compromised by reduced clinical efficacy [9].

Given these limitations, guanidines emerge as promising scaffolds for new therapeutic alternatives. Present in natural products and established drugs such as metformin and guanabenz, these compounds exhibit excellent structural versatility, enabling pharmacological optimization. Moreover, they present a broad spectrum of activity, including anti-bacterial and anti-parasitic properties, acting on specific targets with reduced host toxicity [10,11,12].

LQOF-G2 is a synthetic guanidine compound whose leishmanicidal potential has been previously investigated by our group. In in vitro assays against *Leishmania* (*Viannia*) *braziliensis*, LQOF-G2 stood out among seven compounds by demonstrating significant activity against intracellular amastigotes, with an EC_50_ of 4.2 ± 0.5 µmol/L and a high selectivity index (SI = 38.9). Further, the compound’s anti-amastigote efficacy was associated with alterations in host cell responses, characterized by increased production of nitric oxide (NO), reactive oxygen species (ROS), and TNF-α, along with a reduction in anti-inflammatory cytokines like IL-10. In parallel, the absence of tissue toxicity in preliminary models indicated a favorable safety profile for further investigation [13].

Considering the promising in vitro results, a transition to preclinical models is essential to confirm the potential of LQOF-G2. In this context, our study evaluates the in vivo efficacy of LQOF-G2, characterizes treatment-associated changes in the host immune response in hamsters infected with *L.* (*V.*) *braziliensis*, and assesses its pharmacokinetic profile.

## 2. Results

### 2.1. Evaluation of the Effect of LQOF-G2 Treatment on Lesion Size and Body Weight in Hamsters Infected with L. (V.) braziliensis

The therapeutic effect of LQOF-G2 was initially evaluated by monitoring the progression of cutaneous lesions in golden hamsters infected with *L.* (*V.*) *braziliensis*. As shown in Figure 1A, the control group exhibited a marked and progressive increase in lesion size over the eight-week experimental period. In contrast, animals treated with LQOF-G2 (5 and 10 mg/kg) showed a significant reduction in lesion progression starting from the sixth week post-infection (i.e., two weeks after treatment initiation).

At the 5 mg/kg dose, lesion size was reduced by 25.9 ± 1.9% at week 6, 55.6 ± 3.2% at week 7, and 76.5 ± 5.9% at week 8 (*p* < 0.05) compared with the control group. Similarly, treatment with 10 mg/kg resulted in reductions of 33.3 ± 3.1%, 52.8 ± 4.7%, and 64.7 ± 5.2% at the same time points, respectively (*p* < 0.05). Notably, both LQOF-G2 doses demonstrated lesion resolution efficacy like that observed with the reference drug, Glucantime (50 mg/kg), at the end of the experiment (Figure 1A).

Systemic safety was assessed through weekly monitoring of body weight (Figure 1B). A gradual and consistent increase in body weight was observed across all experimental groups throughout the study. No statistically significant differences were detected between LQOF-G2-treated groups and the control group, indicating that the compound, at the tested doses, did not induce overt toxicity affecting normal weight gain.

### 2.2. Evaluation of Parasite Load in LQOF-G2-Treated Hamsters Infected with L. (V.) braziliensis

Parasite burden was assessed at the end of the therapeutic protocol using a limiting dilution assay. At the lesion site, treatment with LQOF-G2 at 5 mg/kg resulted in a significant reduction of 82.2 ± 8.9% (*p* < 0.001) in parasite density (Figure 2A). An even more pronounced effect was observed at 10 mg/kg, with a reduction of 98.6 ± 0.8% (*p* < 0.001) compared to the untreated control group. Consistent results were observed in the draining lymph nodes (Figure 2B). Both tested doses of LQOF-G2 led to substantial reductions in parasite burden, reaching 98.7 ± 1.4% (*p* < 0.001) at 5 mg/kg and 99.7 ± 0.3% (*p* < 0.001) at 10 mg/kg relative to controls. Notably, the reductions in parasite burden observed with LQOF-G2 were numerically similar to those observed with the reference drug, Glucantime, under the experimental conditions evaluated, highlighting the strong leishmanicidal potential of this compound in the in vivo model (Figure 2).

### 2.3. Evaluation of LQOF-G2 In Vivo Toxicity

The biological safety of systemic treatment with LQOF-G2 was assessed through clinical, biochemical, and hematological analyses after four weeks of daily administration. As shown in Table 1, evaluation of hepatic integrity markers revealed that the infected control group exhibited significantly elevated serum AST and ALT levels compared to healthy animals (*p* < 0.05), indicating hepatocellular damage associated with disease progression.

Treatment with LQOF-G2 demonstrated a favorable profile: at 10 mg/kg, the compound significantly reduced AST levels, bringing them closer to those observed in non-infected animals. In contrast, the reference drug Glucantime (50 mg/kg) resulted in even higher AST levels than those observed in the infected control group (*p* < 0.05), suggesting a potential hepatotoxic effect associated with antimonial therapy.

Regarding renal function, urea levels remained stable across all groups. However, increased creatinine levels were observed in both the infected control and Glucantime-treated groups. Notably, treatment with LQOF-G2 at 5 mg/kg resulted in lower creatinine levels, although this numerical reduction was not statistically significant compared to the control group.

Hematological evaluation (Table 2) showed that LQOF-G2 treatment did not induce significant changes in erythrocyte parameters, including red blood cell count, hemoglobin levels, and hematocrit. Additionally, erythrocyte indices (MCV, MCH, and MCHC) and leukocyte profiles (total and differential counts) remained within normal ranges and were comparable to those of healthy animals. These findings indicated the absence of myelotoxicity or severe immunosuppression at the tested therapeutic doses.

### 2.4. Estimation of Cytokine Production

The cytokine response associated with LQOF-G2 treatment was evaluated by measuring cytokine and nitrite levels in the supernatants of Leishmania antigen-stimulated lymph-node cells obtained from infected animals (Figure 3). Compared to healthy animals (S), the infected control group (C) exhibited elevated levels of TNF-α, IFN-γ, IL-12, IL-10, and IL-6, consistent with an infection-associated immune response. As shown in Figure 3, treatment with LQOF-G2 significantly increased TNF-α levels at the 10 mg/kg dose compared to the control group (*p* < 0.001). Similarly, IFN-γ levels were significantly elevated at this dose (*p* < 0.001). In contrast, IL-10 levels were significantly reduced in treated groups, with the most pronounced decrease observed at 10 mg/kg (*p* < 0.001) relative to controls.

At 5 mg/kg, LQOF-G2 also induced a significant increase in IFN-γ accompanied by a significant reduction in IL-10, a pattern likewise observed in the Glucantime-treated group. For IL-12 and IL-6, variations were observed among experimental groups; however, these differences did not reach statistical significance. Similarly, nitrite (nitric oxide) levels remained comparable across groups, with no significant differences detected.

### 2.5. Pharmacokinetic Studies

#### 2.5.1. Tolerability and Observations

No clinical signs of toxicity or mortality were observed following either intravenous (IV) or intraperitoneal (IP) administration. Dosing accuracy remained within 5% of the target across all groups. All planned sampling time points were successfully executed.

#### 2.5.2. Plasma Concentration–Time Profiles

Following intravenous (IV) administration at 5 mg/kg, plasma concentrations declined in a biphasic manner and were characterized by a rapid distribution phase followed by a slower elimination phase (Figure 4), with relatively limited inter-animal variability beyond the initial time points (Figure 5). The extrapolated C_0_ was 6823 ± 1104 ng/mL, with a systemic clearance of 46 ± 4 mL/min/kg and a steady-state volume of distribution (Vdss) of 11 ± 1 L/kg. The terminal half-life was 6.8 ± 0.4 h, and the corresponding AUC_0–∞_ was 1810 ± 135 ng·h/mL (Table 3). Percent extrapolation to infinity was low (3.2–3.6%), indicating that AUC_0–∞_ was well supported by the observed concentration–time data and that AUC_0–t_ captured most of the exposure (Table 4).

Following intraperitoneal (IP) administration at 10 mg/kg, rapid systemic absorption was observed, with quantifiable plasma concentrations detected as early as 15 min (Figure 5). Nevertheless, plasma concentrations were more dispersed over time, indicating greater variability in absorption and/or terminal exposure among animals, particularly during the later sampling period (Figure 6). A Cmax of 758 ± 61 ng/mL was reached at 0.25 h (Table 3). The terminal half-life (7.3 ± 1.2 h) was similar to that observed following IV administration, indicating elimination kinetics independent of the route of administration (Table 3). Systemic exposure (AUC_0–∞_ = 1580 ± 133 ng·h/mL) corresponded to an absolute bioavailability of 44 ± 4% (Table 3). Bioavailability after IP dosing showed moderate inter-animal variability, with values ranging from 39% to 46% based on AUCinf and from 39% to 43% based on AUC_0–t_. This indicates a relatively consistent, though not identical, systemic exposure after IP administration (Table 4).

For the IP route, percent extrapolation was moderate (5.0–10.6%), indicating that AUC_0–∞_ was well supported by the observed data (Table 4).

Dose-normalized AUC_0–∞_ was higher following intravenous administration of 5 mg/kg than following intraperitoneal administration of 10 mg/kg. However, because dose and administration route differed between groups, this comparison was interpreted descriptively and was not used to infer dose proportionality (Figure 6).

## 3. Discussion

LQOF-G2, a guanidine derivative with previously demonstrated antileishmanial activity in vitro [13], represents a promising scaffold for new treatments against leishmaniasis. Considering that transitioning from cellular assays to complex biological systems is a critical step in validating new bioactive compounds [14,15], this study characterized the impact of LQOF-G2 on infection progression in golden hamsters infected with *L.* (*V.*) *braziliensis*, as well as its pharmacokinetic profile. This integrated approach provides complementary efficacy and pharmacokinetic information for the preclinical characterization of LQOF-G2. However, because the efficacy and pharmacokinetic studies were conducted in different animal species and under different physiological conditions, the PK findings should not be interpreted as a direct determinant of the therapeutic response observed in the hamster model.

The efficacy study was conducted in female golden hamsters infected with *L.* (*V.*) *braziliensis*, a model selected because this parasite species generally produces limited or self-resolving disease in conventional mouse strains, whereas hamsters provide a more sustained cutaneous infection suitable for therapeutic evaluation [16]. In contrast, pharmacokinetic characterization was performed in healthy male BALB/c mice within an established preclinical PK platform. Therefore, species-, sex-, and disease-related differences may influence LQOF-G2 disposition, and pharmacokinetic parameters obtained in mice cannot be directly extrapolated to infected hamsters.

In this study, experiments began by evaluating LQOF-G2 on the progression of ear lesions in golden hamsters. While the control group exhibited continuous, marked lesion progression, treatment with LQOF-G2 at 5 and 10 mg/kg significantly altered lesion progression over time, with significant differences compared with the control group becoming evident from week 6 post-infection. Notably, the efficacy of LQOF-G2 was similar to that observed with the standard dose of Glucantime (50 mg/kg). In parallel, steady weight gain across all groups suggests that the tested doses did not cause overt toxicity affecting general physiological status.

Beyond external lesion resolution, a new leishmanicidal agent must control parasite burden in deeper tissues. It is important to determine whether LQOF-G2 activity is restricted to the inoculation site or is also associated with reductions in parasite burden at distant sites, such as draining lymph nodes. The lymphatic system plays a central role in the pathogenesis of cutaneous leishmaniasis, acting in both immune response orchestration and parasite persistence. In chronic models, eliminating parasite burden in lymph nodes represents a major therapeutic challenge, often persisting after apparent lesion resolution [17]. Our results demonstrated that LQOF-G2 promoted a marked reduction in parasite density both at the lesion site and in draining lymph nodes, showing efficacy comparable to the reference drug even at 5 mg/kg. Notably, the 10 mg/kg dose resulted in a 99.7% reduction in lymph node parasite burden, with no statistically significant difference detected compared with Glucantime (50 mg/kg). The marked reduction in parasite burden at both the lesion site and draining lymph nodes demonstrates in vivo antileishmanial activity beyond the primary infection site, consistent with established efficacy indicators in relevant models [18,19], although the underlying tissue exposure to LQOF-G2 was not directly determined.

Following efficacy confirmation, the systemic safety of LQOF-G2 was evaluated through biochemical markers. In the infected control group, a pronounced elevation in transaminases (AST and ALT) was observed compared to healthy animals, suggesting that *L.* (*V.*) *braziliensis* infection can induce hepatocellular injury [20,21]. This is consistent with the disease’s pathophysiology, where parasite dissemination and systemic inflammatory responses compromise hepatic parenchyma integrity. Glucantime-treated animals exhibited the highest AST levels, exceeding those of the control group [22]. This reinforces the intrinsic hepatotoxic profile of pentavalent antimonials, whose mechanisms are associated with oxidative stress, thiol depletion, and mitochondrial dysfunction [12,23]. These findings support further investigation of LQOF-G2 while highlighting the need for more comprehensive toxicological assessment.

Regarding renal function, urea levels remained stable across groups. Although creatinine levels were numerically lower in the LQOF-G2 5 mg/kg group than in the infected control group, this difference was not statistically significant. Therefore, the present findings do not provide evidence of a treatment-related renal adverse effect based on the biochemical parameters evaluated.

Hematological parameters provided additional information supporting the tolerability of LQOF-G2 under the experimental conditions evaluated. No significant changes were observed in erythrocyte parameters, including red blood cell count, hemoglobin levels, and hematocrit, following treatment. Erythrocyte indices, including MCV, MCH, and MCHC, as well as total and differential leukocyte counts, remained comparable across the experimental groups. These findings do not indicate marked hematological alterations or overt myelotoxicity at the tested therapeutic doses. However, the hematological findings should be interpreted within the scope of the parameters evaluated, as more comprehensive toxicological investigations, including histopathological assessment, are required to establish the systemic safety profile of LQOF-G2.

The safety findings of the present study complement our previous toxicological characterization of LQOF-G2 [13]. In that study, LQOF-G2 showed no relevant cytotoxicity in mammalian cell models, including RAW 264.7, HEK-293, Caco-2, and A549 cells, at the concentrations evaluated. Ex vivo studies using isolated guinea pig tissues also showed no significant effects on papillary muscle contractility or vasodilator activity in aortic rings under the tested conditions, although pharmacological effects were observed in other preparations at higher concentrations. Together, these findings provide complementary evidence supporting the tolerability of LQOF-G2 across different experimental systems. Nevertheless, the absence of histopathological evaluation in the present study limits the assessment of potential organ-specific toxicity. Therefore, comprehensive histopathological and toxicological studies will be necessary to further establish the safety profile of LQOF-G2.

Following therapeutic confirmation, treatment-associated cytokine responses were assessed in lymph-node cell cultures following ex vivo stimulation with *L.* (*V.*) *braziliensis* antigen. LQOF-G2 treatment was associated with increased TNF-α and IFN-γ responses and reduced IL-10 levels compared with infected untreated animals. This cytokine pattern is consistent with a more pro-inflammatory/type-1-associated immune response [24,25]. However, because the cultures consisted of unfractionated lymph-node cells, the cellular populations responsible for these cytokine responses were not characterized. Therefore, these findings should not be interpreted as direct evidence of a specific Th1 or Th2 cellular response or as demonstrating a Th1/Th2 shift. Moreover, because murine ELISA kits and recombinant murine standards were used for cytokine determination in golden hamsters without independent species-specific analytical validation, the absolute cytokine concentrations should be interpreted cautiously. Accordingly, the cytokine findings should be regarded as exploratory and supportive rather than quantitatively validated evidence of immunomodulation, and primarily as treatment-associated differences observed under the same assay conditions. Further studies using hamster-specific reagents and cellular phenotyping will be necessary to quantitatively confirm these findings and characterize the underlying immune mechanisms.

A well-established effector mechanism associated with type-1 inflammatory responses is the induction of inducible nitric oxide synthase (iNOS), which can contribute to intracellular parasite killing. In the present study, however, LQOF-G2 did not significantly alter nitrite levels, suggesting that changes in nitric oxide production were not detected under the experimental conditions evaluated [26,27].

The differences observed between the previous macrophage-based in vitro model and the present ex vivo lymph-node-cell system may reflect differences in cellular composition, host species, experimental context, and stimulation conditions [13]. While the previous study evaluated a relatively homogeneous population of infected macrophages, the present experiments used unfractionated lymph-node cells stimulated with *L.* (*V.*) *braziliensis* antigen, representing a heterogeneous immune-cell response. Therefore, the lack of a significant change in nitrite levels in the present study does not necessarily contradict the increased nitric oxide production previously observed in infected macrophages.

The pharmacokinetic findings provide complementary information on the systemic disposition of LQOF-G2 and contribute to its preclinical characterization. However, the relationship between systemic exposure and drug concentrations at the site of infection remains to be established. The characterization of systemic exposure, disposition, and absorption provides important preliminary information for the further preclinical development of bioactive compounds. This study provides a preliminary pharmacokinetic characterization of LQOF-G2 in mice following intravenous (IV) and intraperitoneal (IP) administration, enabling comparison with existing agents. Overall, LQOF-G2 exhibited measurable systemic exposure, moderate clearance, a prolonged terminal half-life, and moderate absolute bioavailability following intraperitoneal administration. These findings provide supportive pharmacokinetic information for further preclinical investigation of LQOF-G2.

Following IV administration at 5 mg/kg, LQOF-G2 achieved a high initial plasma concentration and a prolonged terminal half-life. The clearance (46 ± 4 mL/min/kg) indicates a moderate rate of elimination, corresponding to 40–50% of mouse hepatic blood flow [28]. This clearance is comparable to that reported for miltefosine and emerging azole compounds and lower than that reported for some rapidly eliminated nitroimidazole analogs [29]. The steady-state volume of distribution (Vdss = 11 ± 1 L/kg) was substantially greater than total body water, consistent with extensive apparent distribution beyond the plasma compartment. However, because tissue concentrations were not directly measured, the specific organs, tissues, or cellular compartments contributing to this distribution cannot be determined from the present data.

Systemic exposure after IP administration was substantial, corresponding to an absolute bioavailability of 44 ± 4%. Although moderate, this value matches or exceeds that reported for several investigational antileishmanial agents administered via non-IV routes [30]. The incomplete bioavailability may be attributed to first-pass hepatic metabolism or partial pre-systemic degradation [31]. However, because pharmacokinetic characterization was performed in healthy male BALB/c mice whereas therapeutic efficacy was evaluated in infected female golden hamsters, the present data do not allow a direct assessment of the relationship between systemic exposure and therapeutic response. Overall, these findings provide supportive pharmacokinetic information for further preclinical investigation of LQOF-G2.

From a dosing perspective, the combination of moderate clearance and a terminal half-life of approximately 7 h provides preliminary information on the systemic persistence of LQOF-G2 in mice. However, the present pharmacokinetic study was designed as a single-dose characterization and did not evaluate repeated administration or pharmacokinetic–pharmacodynamic relationships. Therefore, the optimal dosing interval cannot be established from the current data. Further repeated-dose pharmacokinetic and pharmacodynamic studies will be necessary to determine the appropriate dosing interval and to establish the relationship between systemic exposure and therapeutic response.

Overall, the pharmacokinetic findings provide preliminary information on the systemic disposition of LQOF-G2 in mice. Because tissue concentrations were not directly measured and the PK and efficacy studies were conducted in different animal species and physiological conditions, further studies are needed to characterize tissue distribution and establish pharmacokinetic–pharmacodynamic relationships.

## 4. Materials and Methods

### 4.1. Chemicals and Pharmaceuticals

Schneider and Roswell Park Memorial Institute (RPMI 1640) media, Tween 80 (polysorbate 80), and the antibiotic solution (penicillin 10,000 U/mL; streptomycin 10 μg/mL) were purchased from Sigma-Aldrich (St. Louis, MO, USA). Fetal bovine serum (FBS) was obtained from Cultilab (São Paulo, SP, Brazil), and dimethyl sulfoxide (DMSO, 99%) from Mallinckrodt Chemicals (St. Louis, MO, USA). Thiopental (Thiopentax^®^) and meglumine antimoniate (Glucantime^®^) were acquired from Cristália and Aventis Pharma (São Paulo, SP, Brazil), respectively. Sandwich-type ELISA kits for murine cytokines (IL-12, TNF-α, IFN-γ, IL-6, and IL-10) were supplied by PeproTech^®^ (São Paulo, SP, Brazil), while the Panótico Rápido hematological staining kit was obtained from Laborclin (Curitiba, PR, Brazil).

### 4.2. Compound LQOF-G2

LQOF-G2 (Figure 7) was synthesized as previously described [32].

### 4.3. Animals

Golden hamsters (4–5 weeks old, female) were obtained from the Central Animal House of Parnaíba Delta Federal University, Parnaíba, Brazil, and maintained under controlled temperature (21 ± 1 °C; relative humidity 60%) and light conditions (12 h light/dark cycle), with access to food pellets and water ad libitum. Animals were treated according to Resolution number 1000 (2012) of the Federal Council of Veterinary Medicine, Brazil, and all experimental protocols were approved by the Ethics Committee for Animal Research (CEPA) of Federal University of Parnaíba Delta (process number 1804/25).

### 4.4. L. (V.) braziliensis Parasite Strain Maintenance

The protozoan species *Leishmania* (*Viannia*) *braziliensis* (MHOM/BR/1975/M2903) was cultured in supplemented Schneider’s medium (10% fetal bovine serum and 1% antibiotic solution), maintained in a biological oxygen demand (B.O.D.) incubator SolidSteel (São Paulo SP, Brazil) at 26 °C and pH 7 [33]. To preserve infectivity, in vivo passages were performed every 4–6 weeks by inoculation into the ear of female hamsters. Following the development of ulcerative lesions, promastigote forms were recovered by excision of the affected tissue under local anesthesia and prior aseptic preparation of the area, with appropriate hygienic precautions taken throughout the procedure to minimize discomfort and prevent contamination [34]. Subsequently, the promastigotes were re-cultured in supplemented medium for use in subsequent assays.

### 4.5. Efficacy Evaluation of LQOF-G2 in Golden Hamsters Infected with L. (V.) braziliensis

For experimental infection, 1 × 10^6^ stationary-phase promastigotes were subcutaneously inoculated into the ear of female hamsters using 100 μL of phosphate-buffered saline (PBS). Following infection, the animals were randomly assigned to four experimental groups (*n* = 10 per group) using a computer-generated random sequence based on initial body weight: a negative control (NC), treated with vehicle containing PEG200/Solutol HS15/phosphate buffer pH 7.4 (20/5/75%); a positive control (PC), treated with Glucantime^®^ at a dose of 50 mg/kg [35]; and two groups treated with LQOF-G2 at doses of 5 mg/kg and 10 mg/kg, both administered daily via intraperitoneal injection. Additionally, a separate group of healthy animals (*n* = 10), which were neither infected nor treated, was maintained under identical environmental conditions to establish baseline parameters for normal physiological, biochemical, hematological, and immunological ranges.

After four weeks post-infection, after lesion establishment, the treatment protocol was initiated. The animals were treated daily for a duration of exactly four weeks (28 days). Lesion progression was monitored weekly using a micrometer (Mitutoyo Corporation, Tokyo, Japan), with evaluation based on the difference in thickness between the infected ear and the contralateral uninfected ear of the same animal, as an indirect measure of inflammatory progression. The experimental follow-up lasted four weeks, after which the animals were euthanized 24 h following the final therapeutic intervention [34].

To minimize experimental bias in accordance with the ARRIVE 2.0 guidelines, investigators were blinded to group allocations during weekly lesion measurements, sample collection, parasite burden determination, and data analysis.

### 4.6. Parasite Burden-Limiting Dilution Assay

Following euthanasia of the animals subjected to the previously described experimental procedure, the lesioned tissue and draining auricular lymph nodes were collected. The samples were homogenized by maceration in a glass apparatus, followed by successive washes with PBS and centrifugation at 2000× *g* to remove cellular debris. The resulting single-cell suspension was then split into two aliquots; the first aliquot was resuspended in 1 mL of supplemented Schneider’s medium and processed using a limiting dilution assay, consisting of serial tenfold dilutions in quadruplicate (1:10 to 1:10^11^) plated in sterile 96-well plates.

The cultures were incubated at 26 °C for 15 days in a biological oxygen demand (B.O.D.) incubator and subsequently assessed for parasite viability using inverted microscopy (Nikon TS-100, Tokyo, Japan). Positivity was defined by the presence of viable, motile promastigote forms, and the parasitic load was estimated based on the highest dilution that exhibited detectable growth [36].

### 4.7. Ex Vivo Antigen-Stimulated Cytokine Production by Lymph-Node Cells

For cytokine quantification (pg/mL), the second aliquot of cells isolated from the auricular lymph nodes was adjusted to a concentration of 5 × 10^6^ cells/mL and cultured in supplemented RPMI 1640 medium (10% fetal bovine serum and 1% antibiotic solution). The cells were distributed at 1 mL per well into sterile 24-well plates in triplicate and stimulated ex vivo with *Leishmania* (*Viannia*) *braziliensis* antigen at a concentration of 50 μg/mL. The antigen was prepared from 1 × 10^10^ stationary-phase promastigotes, following the methodology previously described [37]. The plates were incubated for 72 h at 37 °C in a humidified atmosphere containing 5% CO_2_. Following incubation, culture supernatants were collected for quantification of TNF-α, IFN-γ, IL-12, IL-10, and IL-6 by enzyme-linked immunosorbent assay (ELISA), according to the manufacturer’s instructions. Cytokine concentrations were determined from standard curves generated using recombinant murine cytokines, with absorbance measured at 450 nm [37].

### 4.8. Toxicity Assessment

As an indicator of treatment safety, a toxicity assessment was conducted through the analysis of clinical and laboratory parameters using the same experimental groups of golden hamsters described in Section 4.5 (*n* = 10 per group, receiving daily i.p. doses of vehicle, Glucantime, or LQOF-G2 at 5 and 10 mg/kg for 4 weeks). Animals were monitored weekly for potential alterations, including alopecia, changes in body weight, occurrence of secondary skin lesions, and mortality. For laboratory evaluations, serum samples and heparinized whole blood were obtained immediately after euthanasia, which was performed 24 h following the final therapeutic intervention.

For biochemical analysis, serum samples were used to quantify urea and creatinine levels to evaluate renal function, as well as the activities of alanine aminotransferase (ALT) and aspartate aminotransferase (AST), which serve as indicators of hepatic toxicity. These measurements were performed using a Cobas Mira Plus^®^ analyzer (Roche Diagnostic System, São Paulo, Brazil).

In parallel, hematological evaluation was carried out using heparinized whole blood, processed immediately after collection to prevent hemolysis. This included measurements of hemoglobin (Hb), erythrocyte count (RBC), hematocrit (Hct), and erythrocyte indices such as mean corpuscular volume (MCV), mean corpuscular hemoglobin (MCH), and mean corpuscular hemoglobin concentration (MCHC). Additionally, total and differential leukocyte counts were performed to assess inflammatory responses or treatment-induced immunosuppression, using a Hematoclin 2.8 VET analyzer (Bioclin, São Paulo, Brazil) [37].

### 4.9. Pharmacokinetic Studies

#### 4.9.1. Animals

All experiments were performed at Evotec France SAS’s facility, authorized by the French Competent Authority (Authorization # D31 555 09) under the APAFIS#04.932.02 Animal Use Project and reviewed by the institutional ethics committee (CEPAL- #: CE 029) and the internal Animal Welfare Body (SBEA). Evotec France SAS’s animal use and care program (Institution #: 001433) is accredited by AAALAC International.

Male Balb/cByJRj mice [38] aged 11 weeks, provided by Janvier Labs, Le Genest-Saint-Isle, France, were housed in a barrier facility, maintained at controlled temperature and humidity with ad libitum access to food and filtered water.

#### 4.9.2. Test Article and Formulations

LQOF-G2 (MW 407.6 g/mol) was formulated in PEG200/Solutol HS15/phosphate buffer pH 7.4 (20/5/75%) and prepared freshly on each day of dosing. The final nominal concentrations were 1 mg/mL for intravenous (IV) dosing and 2 mg/mL for intraperitoneal (IP) dosing. Formulations were analytically confirmed to be 95–97% of nominal concentrations.

#### 4.9.3. Dosing and Experimental Design

The study was conducted in two groups of 3 mice. The first group received LQOF-G2 at 5 mg/kg by IV route at the tail vein (while awake) with an administration volume of 5 mL/kg [39].

The second group received LQOF-G2 at 10 mg/kg by IP route at the lower right quadrant of the abdomen (while awake) with an administration volume of 5 mL/kg. Animals were randomized into dosing groups prior to administration using a computer-generated random sequence based on body weight [39].

#### 4.9.4. Blood Sampling and Processing Collection

Blood was serially withdrawn from each mouse by microsampling technique in K3-EDTA [40]. Blood samples (20 µL at each timepoint) were collected at 0.083, 0.25, 0.5, 1, 2, 4, 7 and 24 h post- IV dose, and collected at 0.25, 0.5, 1, 2, 3, 4, 7, and 24 h post- IP dose. Plasma was separated by centrifugation at 10,000× *g* for 5 min at 4 °C and then stored in 96 well plates at −20 °C until analysis.

#### 4.9.5. Bioanalytical Quantification in Plasma

Plasma concentrations of compound LQOF G2 were determined using a validated LC–MS/MS method [41]. Samples were prepared by protein precipitation with acetonitrile (ACN) containing trifluperidol as the internal standard. Quantification was performed using an LC–MS/MS system (TSQ Altis) (Thermo Fisher Scientific, Waltham, MA, USA).

The assay exhibited a lower limit of quantification (LLOQ) of 2.5 ng/mL, with calibration curves ranging from 2.5 to 5000 ng/mL. Quality control samples met predefined accuracy and precision acceptance criteria.

#### 4.9.6. Pharmacokinetic Calculations

Non-compartmental analysis (Phoenix WinNonLin 7.0) [42] was used to calculate C0, Cmax, Tmax, AUC_0–t_, AUC_0–∞_, apparent clearance (CL/F), apparent volume of distribution (Vd/F), terminal half-life (t1/2), and bioavailability.

### 4.10. Statistical Analysis

Longitudinal lesion-size and body-weight data were analyzed using a mixed-effects model, with treatment group and time as fixed effects and animal as a random effect, followed by Šídák’s multiple-comparisons test. This approach accounted for the repeated measurements obtained from the same animals over time and allowed the inclusion of animals with incomplete longitudinal observations. For other experimental endpoints, differences between groups were evaluated using one-way ANOVA followed by Bonferroni’s post hoc test, or Student’s *t*-test or Mann–Whitney U test, as appropriate. Statistical analyses were performed using GraphPad Prism^®^ version 8.0.

## 5. Conclusions

Our findings support the in vivo antileishmanial activity of LQOF-G2 in a golden hamster model of cutaneous leishmaniasis caused by *Leishmania* (*Viannia*) *braziliensis*. Treatment with LQOF-G2 significantly reduced lesion size and parasite burden at both the infection site and in the draining lymph nodes, with no statistically significant differences detected compared with Glucantime under the experimental conditions evaluated. No marked alterations were observed in the biochemical and hematological parameters evaluated at the tested doses.

Treatment was associated with increased TNF-α and IFN-γ responses and reduced IL-10 levels following ex vivo antigen stimulation, consistent with a more pro-inflammatory/type-1-associated cytokine profile. However, because murine cytokine ELISA reagents and recombinant murine standards were used with golden hamster samples without independent species-specific analytical validation, these findings should be considered exploratory and supportive rather than quantitatively validated evidence of an immunomodulatory mechanism. In addition, the use of unfractionated lymph-node cells does not allow attribution of these responses to specific immune-cell populations.

Pharmacokinetic characterization in male BALB/c mice showed measurable systemic exposure, moderate clearance, a terminal elimination half-life of approximately 7 h, and moderate absolute bioavailability following intraperitoneal administration. These findings provide supportive preliminary pharmacokinetic information for further preclinical investigation; however, the relationship between systemic exposure and therapeutic efficacy in the infected hamster model remains to be established.

Overall, the present results support LQOF-G2 as a candidate for further preclinical investigation in cutaneous leishmaniasis. Additional studies are required to confirm the cytokine findings using hamster-specific or independently validated assays, characterize the cellular mechanisms underlying the observed immune responses, establish tissue and intracellular drug distribution, and define pharmacokinetic–pharmacodynamic relationships and optimal dosing regimens.

## Figures and Tables

**Figure 1:**
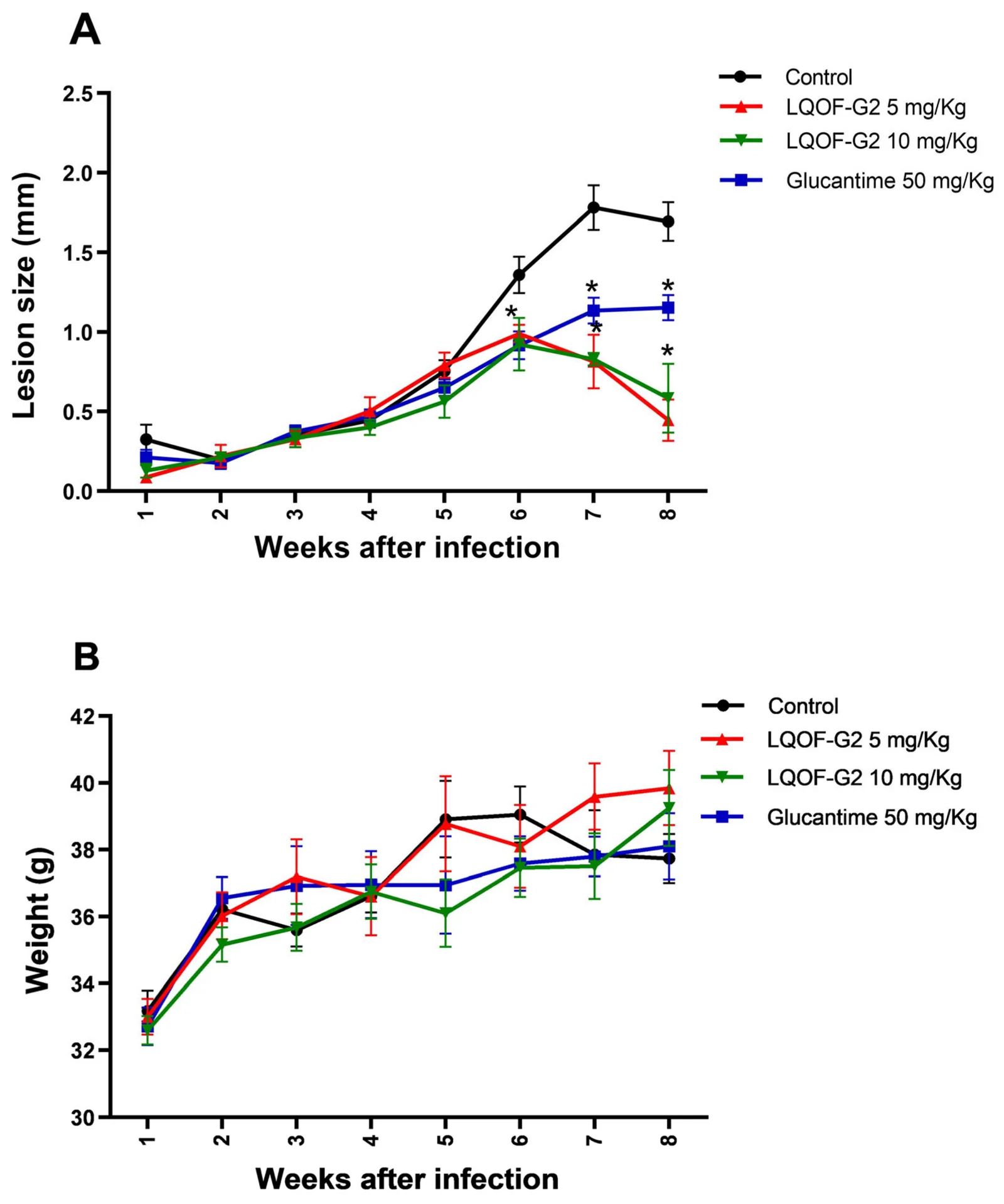
Effect of treatment with LQOF-G2 on lesion size (**A**) and body weight (**B**) in golden hamsters infected with *Leishmania* (*Viannia*) *braziliensis*. Golden hamsters (*n* = 10) were infected in the ear with 1 × 10^6^ stationary-phase promastigotes. After four weeks, animals received intraperitoneal treatment with LQOF-G2 (5 or 10 mg/kg) for four weeks. A separate group was treated with the reference drug (50 mg/kg) over the same period, while the control group received vehicle [PEG200/Solutol HS15/phosphate buffer pH 7.4 (20/5/75%)]. Lesion progression was monitored weekly up to week 8 post-infection. Data are presented as mean ± SEM. Longitudinal lesion-size and body-weight data were analyzed using a mixed-effects model, with treatment group and time as fixed effects and animal as a random effect, followed by Šídák’s multiple-comparisons test. (*) *p* < 0.05 vs. vehicle-treated control.

**Figure 2:**
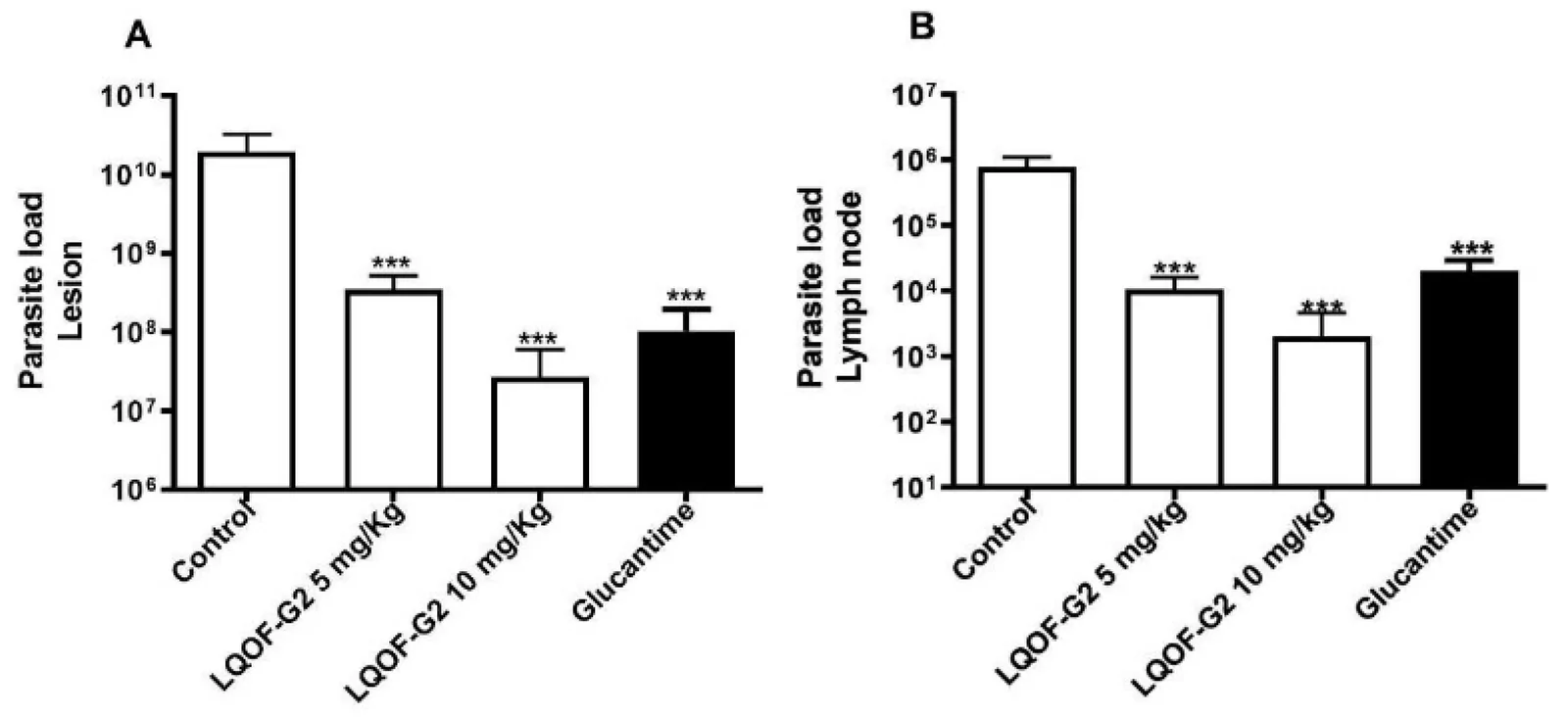
Parasite burden in golden hamsters infected with *L.* (*V.*) *braziliensis* and treated with LQOF-G2. (**A**) Parasite burden at the lesion site; (**B**) parasite burden in the draining lymph node. Golden hamsters (*n* = 10) were infected in the ear with 1 × 10^6^ stationary-phase promastigotes. After four weeks, animals received intraperitoneal treatment with LQOF-G2 (5 or 10 mg/kg) for four weeks. A separate group was treated with the reference drug (50 mg/kg) over the same period, while the control group received vehicle (PEG200/Solutol HS15/phosphate buffer pH 7.4 (20/5/75%)). Parasite load in the lesion and draining lymph node was determined by limiting dilution after euthanasia at the end of treatment. Data are presented as mean ± SEM of parasite burden. (***) *p* < 0.001 vs. control.

**Figure 3:**
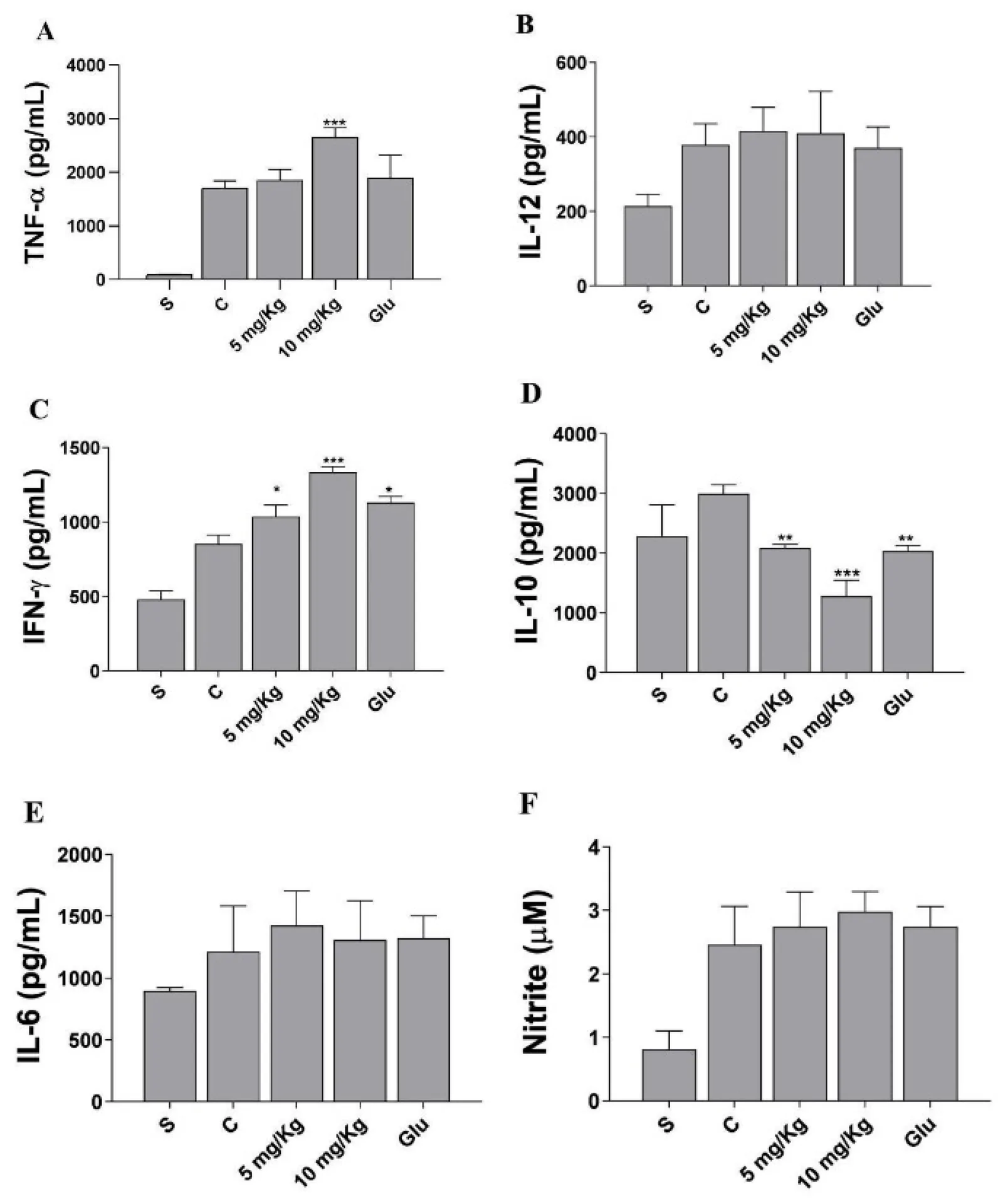
Levels of cytokines and nitric oxide (NO) produced by lymph node cells from golden hamsters infected with *L.* (*V.*) *braziliensis* and treated intraperitoneally with LQOF-G2. Cell suspensions were obtained from lymph nodes at 8 weeks post-infection and stimulated ex vivo with *L.* (*V.*) *braziliensis* antigen (50 μg/mL). Concentrations of TNF-α (**A**), IL-12 (**B**), IFN-γ (**C**), IL-10 (**D**), IL-6 (**E**), and NO (**F**) were measured in culture supernatants by ELISA. Data are presented as mean ± SEM. of parasite load, (*) *p* < 0.05 vs. control; (**) *p* < 0.01 vs. control; (***) *p* < 0.001 vs. control. S: healthy animals; C: control; Glu: Glucantime.

**Figure 4:**
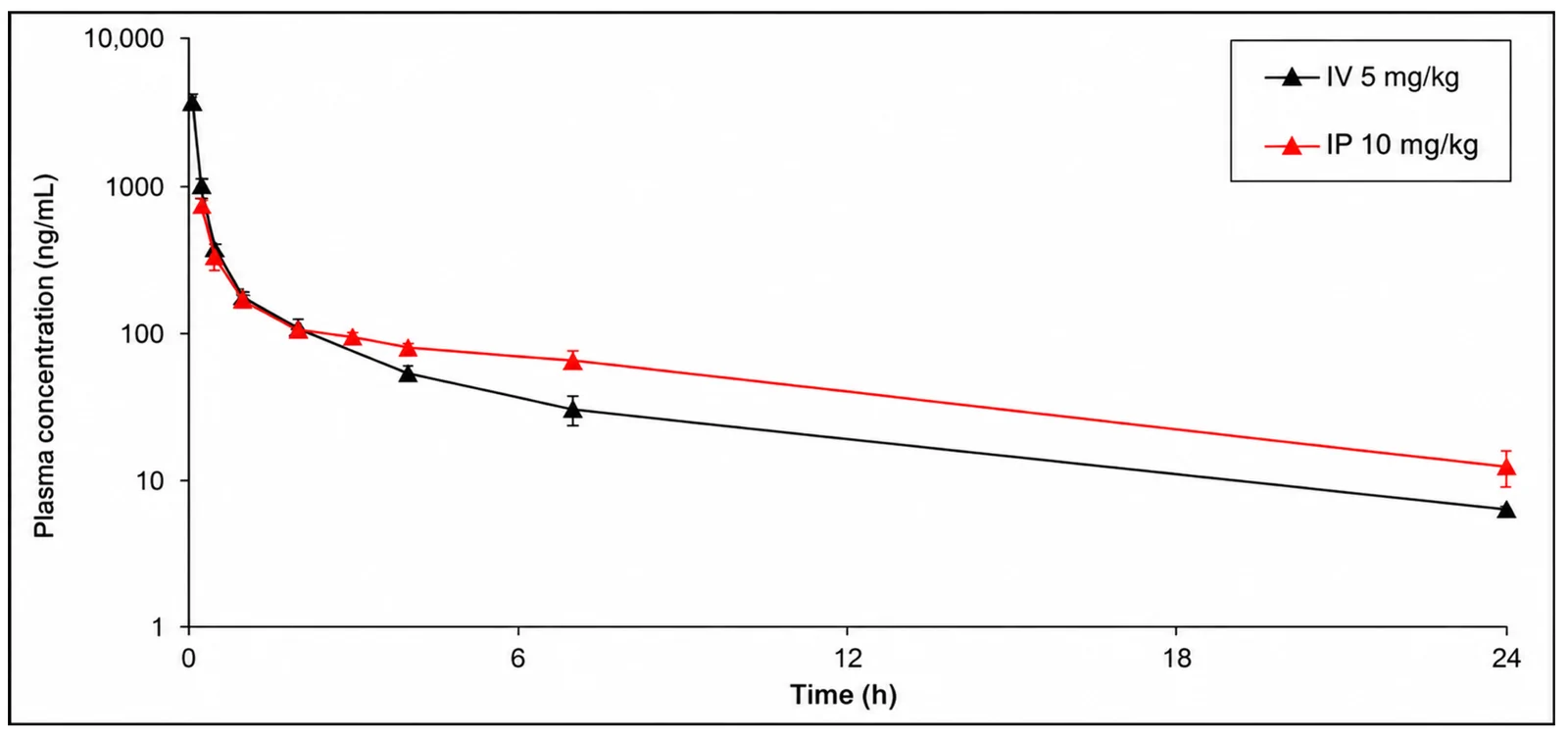
Mean (± SD) plasma concentration–time profiles of LQOF G2 following IV and IP administration in mice. Plasma concentration–time curves for IV (5 mg/kg; black triangles) and IP (10 mg/kg; red triangles) administration in male Balb/cByJRj mice (*n* = 3 per group). Data are presented on a semi-logarithmic scale, illustrating rapid distribution and prolonged elimination following IV dosing, and rapid absorption with moderate Cmax following IP dosing.

**Figure 5:**
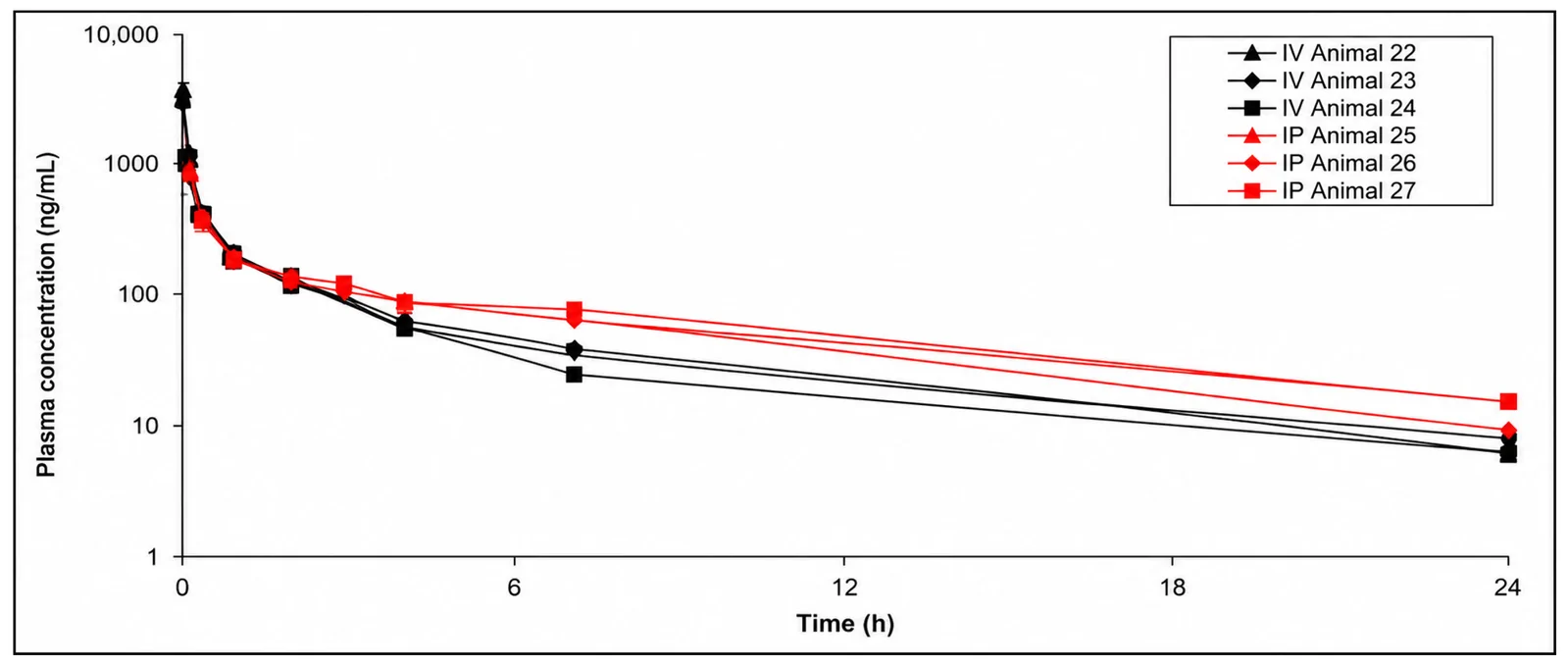
Individual plasma concentration–time profiles of LQOF G2 following IV and IP administration in mice. Individual curves for IV (5 mg/kg; black symbols) and IP (10 mg/kg; red symbols) administration (*n* = 3 per group) are shown on a semi-logarithmic scale, demonstrating low inter-individual variability, rapid distribution and prolonged elimination after IV dosing, and rapid absorption with moderate Cmax after IP dosing.

**Figure 6:**
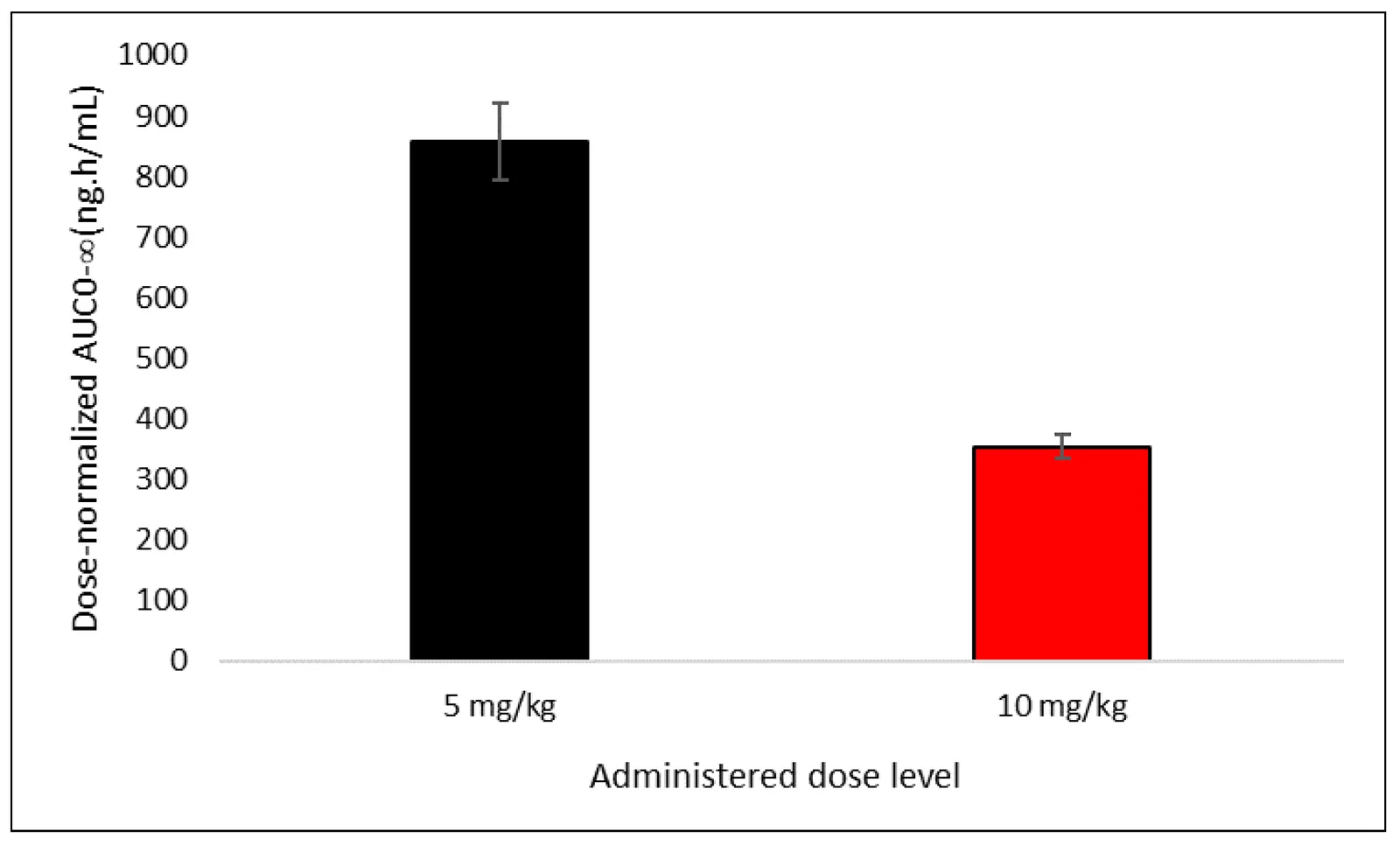
Dose-normalized systemic exposure and relative bioavailability of LQOF-G2 following intravenous and intraperitoneal administration in mice.

**Figure 7:**
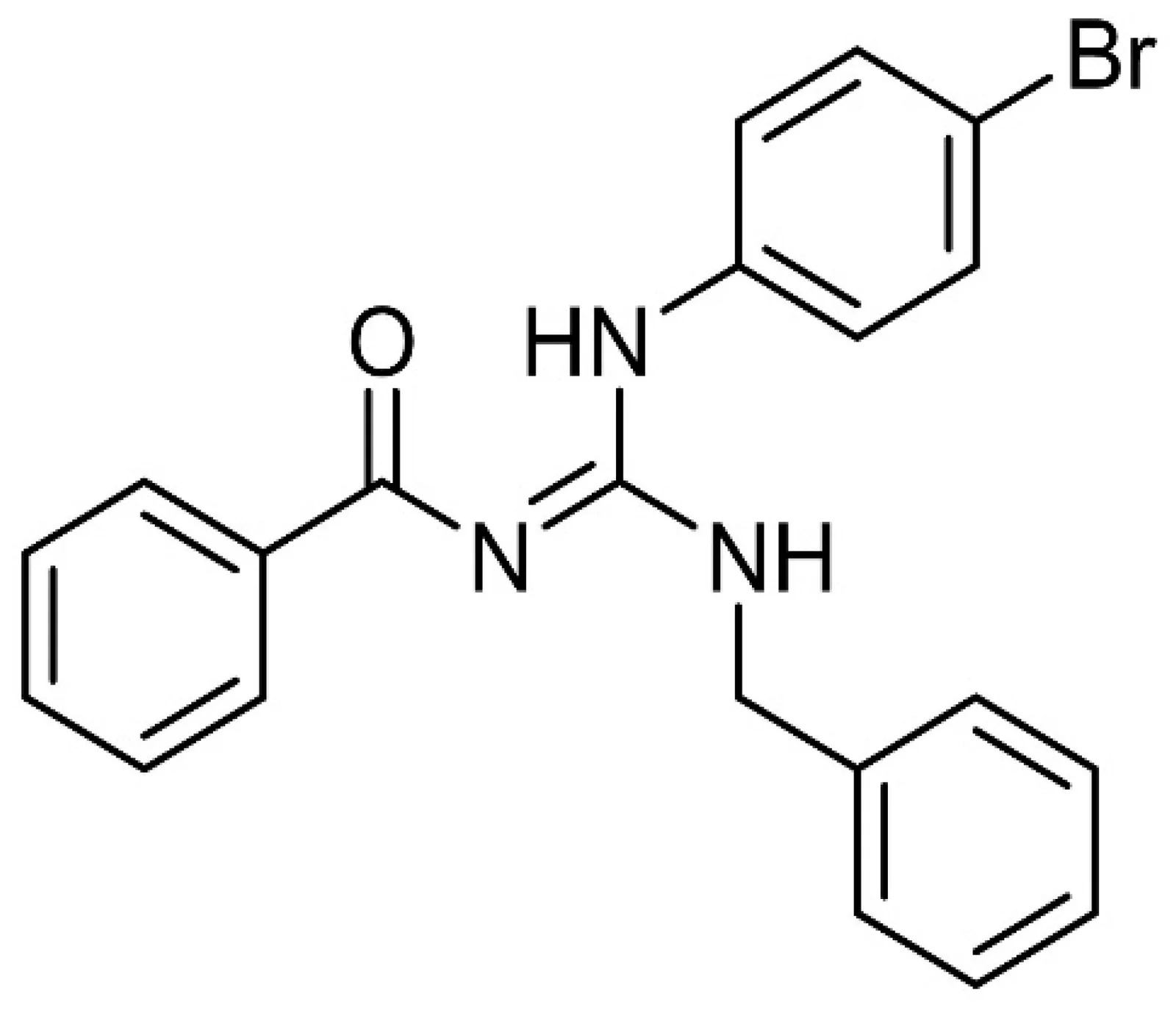
Structure and spectroscopic data (NMR, HRESIMS), purity, and melting point of LQOF-G2. *(Z)-N-((benzylamino)((4-bromophenyl)amino)methylene)benzamide.* ^1^H NMR (400.13 MHz, CDCl_3_, 60 °C) δ (ppm): 12.15 (s, 1H), 8.29 (d, 2H), 7.54–7.48 (m, 5H), 7.41–7.35 (m, 5H), 7.14 (d, 2H), 5.45 (t, 1H), 4.85 (d, 2H). ^13^C NMR (100.61 MHz, CDCl_3_, 60 °C) δ (ppm): 177.5 (C=O), 158.1 (C=N), 138.2 (C), 137.6 (C), 134.1 (C), 133.1 (2CH), 131.6 (p-CH), 128.9 (2CH), 128.8 (2CH), 128.1 (2CH), 127.7 (2CH, p-CH), 127.4 (2CH), 120.6 (C–Br), 44.8 (N–CH_2_). HRESIMS m/z: 408.0701 [M+H]^+^ (calcd for C_21_H_19_BrN_3_O^+^, 408.0706; Δ = 1.2 ppm). Retention time: 37.52 min; purity: 96.5% (HPLC). Melting point: 94.8–96.1 °C.

**Table 1:** Effects of LQOF-G2 on biochemical parameters in peripheral blood of golden hamsters (*n* = 10) subjected to daily treatment for four weeks. Data are presented as mean ± SEM and were analyzed by ANOVA followed by Bonferroni’s test. (a) *p* < 0.05 vs. control; (b) *p* < 0.05 vs. healthy animals.

Parameter	Healthy Animals	Control	Glucantime 50 mg/Kg	LQOF-G2	
				5 mg/Kg	10 mg/Kg
AST (U/L)	50.30 ± 3.20	91.20 ± 4.20 ^b^	113.20 ± 5.20 ^a,b^	81.50 ± 1.70 ^b^	63.60 ± 6.70 ^a^
ALT (U/L)	1.60 ± 0.30	9.10 ± 3.41 ^b^	5.90 ± 0.80 ^b^	8.20 ± 0.04 ^b^	6.80 ± 0.03 ^b^
Urea (mg/dL)	22.10 ± 1.10	25.05 ± 1.70	26.25 ± 1.50	27.92 ± 1.40	18.20 ± 0.80
Creatinine (mg/dL)	0.13 ± 0.03	0.59 ± 0.01	0.69 ± 0.02	0.30 ± 0.07	0.71 ± 0.01

**Table 2:** Effects of LQOF-G2 on hematological parameters in peripheral blood of golden hamsters (*n* = 10) subjected to daily treatment for four weeks. Data are presented as mean ± SEM and were analyzed by ANOVA followed by Bonferroni’s test.

Parameter	Healthy Animals	Control	Glucantime 50 mg/Kg	LQOF-G2	
				5 mg/Kg	10 mg/Kg
Red blood cells (10^6^/mm^3^)	11.8 ± 0.9	12.7 ± 0.8	11.1 ± 0.9	12.3 ± 1	11.4 ± 1.2
Hemoglobin (g/dL)	21.7 ± 0.8	26.4 ± 1.2	21.9 ± 1.7	23.6 ± 0.4	20.9 ± 1.1
Hematocrit (%)	65.7 ± 4.9	67.3 ± 0.9	64.3 ± 1.3	65.2 ± 2.1	63.5 ± 3.1
MCV (fm^3^)	51.3 ± 0.2	51.7 ± 0.6	50.5 ± 1.1	51.3 ± 0.7	49.2 ± 0.6
MCH (pg)	17.8 ± 0.5	17.6 ± 0.6	16.8 ± 0.8	17.7± 0.4	16.9 ± 0.5
MCHC (g/dL)	46.2 ± 0.8	46.2 ± 1.1	42.2 ± 1.5	45.1 ± 1.3	44.2 ± 1.5
Total Leukocytes (10^3^/mm^3^)	11.4 ± 0.5	12.2 ± 1.3	9.1 ± 2.3	12.1 ± 0.3	11.4 ± 0.3
Lymphocytes	87.2 ± 1.7	88.8 ± 1.2	80.4 ± 1.9	86.5 ± 1.2	81.3 ± 1.7
Neutrophils	7.2 ± 0.5	7.5 ± 0.9	9.3 ± 2.1	7.5 ± 0.5	10.4 ± 1.3
Monocytes	1.4 ± 0.1	1.9 ± 0.2	0.9 ± 0.2	2.0 ± 0.3	2.3 ± 0.3
Eosinophils	0.0 ± 0.0	0.0 ± 0.0	0.0 ± 0.0	0.0 ± 0.0	0.0 ± 0.0

**Table 3:** Mean plasma pharmacokinetic parameters of LQOF G2 following IV (5 mg/kg) and IP (10 mg/kg) administration in male Balb/cByJRj mice.

Parameter	Unit	IV 5 mg/kg (Mean ± SD)	IP 10 mg/kg (Mean ± SD)
Dose	mg/kg	5	10
C_0_/C_max_	ng/mL	6823 ± 1104	758.0 ± 61.3
T_max_	h	—	0.25
t_½_	h	6.8 ± 0.4	7.3 ± 1.2
Clearance (CL)	mL/min/kg	46 ± 4	—
Vdss	L/kg	11 ± 1	—
AUC_0–∞_	ng·h/mL	1810 ± 135	1580 ± 133
AUC_0–t_	ng·h/mL	1747 ± 132	1445 ± 77
Bioavailability (F)	%	—	44 ± 4

Values are expressed as mean ± SD (*n* = 3 per group). IP bioavailability was calculated based on dose-normalized AUC relative to the IV group.

**Table 4:** Individual plasma pharmacokinetic parameters of LQOF G2 following IV (5 mg/kg) and IP (10 mg/kg) administration in male Balb/cByJRj mice.

PK Parameter	IV 5 mg/kg			IP 10 mg/kg		
	22	23	24	25	26	27
Dose (mg/kg)	5	5	5	10	10	10
Dose (µmol/kg)	12	12	12	25	25	25
C_0_/C_max_ (ng/mL)	7807	7031	5629	768	693	814
C_0_/C_max_ (nM)	19,154	17,251	13,811	1884	1699	1997
T_max_ (h)	-	-	-	0.25	0.25	0.25
t_1/2_ (h)	6.4	7.1	6.9	5.9	7.7	8.2
MRT (h)	4.0	4.0	3.8	-	-	-
Vdss (L/kg)	11	11	12	-	-	-
CL/CL_F (mL/min/kg)	44	44	50	-	-	-
AUC_inf_ (ng·hr/mL)	1876	1900	1654	1427	1653	1661
AUC_inf_ (nM·hr)	4603	4660	4058	3501	4057	4075
AUC_0–t_ (ng·hr/mL)	1816	1831	1596	1355	1494	1485
AUC_0–t_ (nM·hr)	4455	4492	3915	3325	3665	3642
% extrapolated AUCinf	3.2	3.6	3.5	5.0	9.6	10.6
C_last_ (ng/mL)	6.54	6.74	5.84	8.39	14.5	14.8
Bioavailability (%) Using AUC_inf_	-	-	-	39%	46%	46%
Bioavailability (%) Using AUC_0–t_	-	-	-	39%	43%	42%
Number of Points used for Lambda z	3	3	3	3	4	3
AUC % Extrapolation to infinity	3.2	3.6	3.5	5.0	9.7	10.6
T_last_ (h)	24	24	24	24	24	24

All pharmacokinetic parameters were derived using non-compartmental analysis (Phoenix WinNonlin 7.0).

## Data Availability

The original contributions presented in this study are included in the article. Further inquiries can be directed to the corresponding authors.

## Abbreviations

The following abbreviations are used in this manuscript:

AAALAC	Association for Assessment and Accreditation of Laboratory Animal Care International
ACN	Acetonitrile
ALT	Alanine Aminotransferase
ANOVA	Analysis of Variance
APAFIS	Autorisation de Projet Utilisant des Animaux à des Fins Scientifiques
AST	Aspartate Aminotransferase
AUC	Area Under the Curve
AUC_0–∞_	Area Under the Plasma Concentration–Time Curve from Time Zero to Infinity
AUC_0–t_	Area Under the Plasma Concentration–Time Curve from Time Zero to the Last Measured Time
B.O.D.	Biological Oxygen Demand
C_0_	Initial Plasma Concentration
CEPA	Ethics Committee for Animal Research
CEPAL	Comité d’Éthique en Expérimentation Animale
CL/F	Apparent Clearance
Cmax	Maximum Plasma Concentration
CO_2_	Carbon Dioxide
DMSO	Dimethyl Sulfoxide
EC_50_	Half-Maximal Effective Concentration
ELISA	Enzyme-Linked Immunosorbent Assay
FBS	Fetal Bovine Serum
Hb	Hemoglobin
Hct	Hematocrit
IFN-γ	Interferon Gamma
IL	Interleukin
IL-6	Interleukin-6
IL-10	Interleukin-10
IL-12	Interleukin-12
iNOS	Inducible Nitric Oxide Synthase
IP	Intraperitoneal
IV	Intravenous
LC–MS/MS	Liquid Chromatography–Tandem Mass Spectrometry
LLOQ	Lower Limit of Quantification
MCH	Mean Corpuscular Hemoglobin
MCHC	Mean Corpuscular Hemoglobin Concentration
MCV	Mean Corpuscular Volume
MW	Molecular Weight
NC	Negative Control
NO	Nitric Oxide
NTDs	Neglected Tropical Diseases
PBS	Phosphate-Buffered Saline
PC	Positive Control
PEG	Polyethylene Glycol
RBC	Red Blood Cell Count
ROS	Reactive Oxygen Species
RPMI	Roswell Park Memorial Institute Medium
SAS	Société par Actions Simplifiée
SI	Selectivity Index
TMB	Tetramethylbenzidine
Tmax	Time to Maximum Plasma Concentration
TNF-α	Tumor Necrosis Factor Alpha
Vd/F	Apparent Volume of Distribution
Vdss	Volume of Distribution at Steady State
WHO	World Health Organization
